# Combined Effects of Resveratrol and Vitamin E From Peanut Seeds and Sprouts on Colorectal Cancer Cells

**DOI:** 10.3389/fphar.2021.760919

**Published:** 2021-11-03

**Authors:** Chunfeng Wang, Na Wang, Na Li, Qiuying Yu, Fangyu Wang

**Affiliations:** ^1^ The First Affiliated Hospital of Zhengzhou University, Zhengzhou, China; ^2^ Zhengzhou Nutrition and Health Food Laboratory, Zhengzhou, China; ^3^ School of Food Science and Technology, Henan Agricultural University, Zhengzhou, China; ^4^ Henan Key Laboratory of Animal Immunology, Henan Academy of Agricultural Sciences, Zhengzhou, China

**Keywords:** resveatrol, vitamim E, HCT-8, apoptosis, BAX and BCL-2, caspase

## Abstract

Resveratrol (RES) and Vitamin E (VE) are anti-cancer active ingredients with relatively high content in peanut seeds and sprouts. This study aimed to determine the synergistic inhibitory effect of RES and VE on colorectal cancer. Using 5-FU as a positive drug control, the effect of RES combined with VE on HCT-8 cells was determined, and cell viability was detected using the cell-counting kit 8 (CCK8) method. Cell morphology changes were observed using optical microscopy. Cell migration ability was evaluated by the scratch test, while cell colonies were determined by the cloning test formation ability. Apoptosis status was assessed by flow cytometry and nuclear staining by DAPI, and the expression level of apoptosis-related proteins was determined by western blotting. Compared with the single component group, the RES combined with VE group significantly inhibited the growth and proliferation of HCT-8 intestinal cancer cells *in vitro*. The RES combined with VE group had a greater impact on cell morphology changes and cell colony formation and significantly reduced cell migration ability and intestinal cancer cell apoptosis (*p* < 0.05). Additionally, combined treatment with RES and VE significantly upregulated the expression of pro-apoptotic proteins BAX, caspase-3, caspase-8, and caspase-9, and downregulated the expression of anti-apoptotic protein BCL-2, compared to the single component treatment. RES combined with VE is effective in promoting intestinal cancer cell apoptosis. This study demonstrated the significant positive synergy of RES and VE on HCT-8 cells, providing a new perspective for more effective use of RES.

## Introduction

Colorectal cancer is one of the most common cancers, and it is the third most common cancer in men and the second in women (van Harten-Gerritsen et al., 2015; Farinetti et al., 2017). In 2018, among all cancers globally, colorectal cancer ranked third in incidence (more than 1.8 million new cases) and second in mortality (more than 860,000 deaths) (Bray et al., 2018). The current standard treatments for colon cancer include surgery, chemotherapy, targeted therapy, and radiotherapy (Zullig et al., 2016). Although cancer treatment has improved considerably in the past decade, the survival rate of patients with metastatic and advanced colon cancer is still low (Pabla et al., 2015). Therefore, it is necessary to focus on the molecular mechanism of colon cancer tumorigenesis to determine specific targets for drug development, to help develop natural and effective anti-cancer drugs (Auclin et al., 2017), and provide a new perspective for solving human anti-cancer problems.

Resveratrol (RES) is a member of the stilbene family and is ubiquitous in some seed plants. RES is a non-flavonoid polyphenol compound primarily found in grapes, peanuts, wine, blueberries, bilberries, dark chocolate, and tea. Like many other plant active ingredients (such as flavonoids and catechins), RES is considered a preventive food ingredient. The health benefits of RES were first emphasized in the early 1990s. As a component of red wine, RES has been touted for its beneficial effects, including a wide range of pharmacological properties such as antioxidant, anti-inflammatory, anti-cancer, and immunomodulatory effects. The understanding of the properties of this non-flavonoid polyphenol compound has led to its use in treating diabetes (Ozturk et al., 2017; Shaito et al., 2020), neurodegenerative diseases (Signorelli and Ghidoni, 2005), cancer (Baur and Sinclair, 2006), aging (Raj et al., 2021), obesity (Rauf et al., 2018), and heart disease (Shammugasamy et al., 2015). Among the various pharmacological effects of RES, the most striking is its anti-tumor effect, which is manifested as an inhibitory effect on the three stages of tumor initiation, promotion, and development. RES can inhibit human lung cancer, breast cancer, gastric cancer, liver cancer, leukemia, and other tumor cells to varying degrees through various mechanisms (Rauf et al., 2018).

Vitamin E (VE) in its natural form is mainly found in foods, including some fruits and vegetables (Shammugasamy et al., 2015). The primary dietary source of VE is vegetable oil. Nuts are a good source of VE, while soybean, sunflower, corn, walnut, cottonseed, palm, and wheat germ oils contain more VE than other oils (more than 50 mg vitamin E/100 g oil). At present, natural or synthetic forms of α-tocopherol are often used as VE supplements, which are very popular and help some people to consume VE. VE has diverse biological activities, including antioxidant, anti-inflammatory, neuroprotective, and cholesterol lowering properties (Jiang, 2014).

RES and VE are two common active substances in food, both of which have the advantages of high safety and no negative effects. Studies have shown that they have important anti-oxidative, anti-inflammatory, and anti-cancer roles. Furthermore, RES and VE have similar effects on some biological processes, suggesting that they may have synergistic or additive effects in preventing inflammation (Fasano et al., 2014). Several studies have found that the combined use of multiple antioxidant efficacy factors has a better antioxidant effect than a single component (Mao et al., 2017). Synergy can effectively conserve resources and greatly improve antioxidant and anti-inflammatory capabilities. Therefore, it is necessary to explore whether RES combined with VE have synergistic anti-oxidative, anti-inflammatory, and anti-cancer effects, as well as to understand their possible mechanism of action.

This study aimed to determine the synergistic inhibitory effects of RES and VE on colorectal cancer cell lines. The cell activity, cell migration ability, apoptotic status of cells, and the expression levels of apoptosis-related proteins were determined under different RES concentrations and VE treatment. This is of significance for the in-depth understanding of the health effect mechanisms of food active ingredients and provides a theoretical reference for further clarifying the interaction relationship between various functional ingredients in the diet.

## Manuscript Formatting

### Extraction of RES and VE

RES extraction from peanut sprouts was conducted according to the method of Hao (Hao et al., 2020). Briefly, 5 g of peanut sprout powder was placed in a 50 ml centrifuge tube containing 40 ml of 85% aqueous ethanol solution. The centrifuge tube was placed in an ultrasonic water bath at 80°C for 45 min with intermittent shaking. After standing overnight, 8 ml of the supernatant was filtered using an alumina chromatography column. Next, the 10 ml filtrate was blown with nitrogen at 40°C until it reached 1 ml. Thereafter, the filtrate was filtered through a 0.22 µm filter membrane and analyzed using high-performance liquid chromatography (HPLC). Follow-up experiments were conducted when the RES content reached 96%.

Vitamin E was extracted from peanuts according to the method of Li (Li et al., 2017). Briefly, 10 g crushed peanuts were placed in a saponification bottle with 30 ml of absolute ethanol. Next, 5 ml of 10% ascorbic acid and 2 ml benzopyrene standard solution were added and thoroughly mixed. The mixture was then refluxed for 30 min in boiling water. After saponification, the mixture was immediately placed into ice water to cool. The saponification bottle and residue were washed twice with approximately 100 ml of ether. Subsequently, the ether layer in the separatory funnel was washed with approximately 50 ml of water, and checked using pH test paper until the water layer was no longer alkaline. After concentration, the supernatant was used for HPLC analysis.

### Cell Cultures

Human colon cancer cell line, HCT-8, was purchased from the Shanghai Institute of Cell Biology, Chinese Academy of Sciences (Shanghai, China). The cells were cultured in 1640 medium [Invitrogen Trading (Shanghai) Co. Ltd., Shanghai, China] containing 10% fetal bovine serum (Sijiqing, Hangzhou, China). All cell lines were cultured in a 5% carbon dioxide incubator [Thermo Fisher Scientific (China) Co., Ltd., Shanghai, China] at 37°C. The cryopreservation and passage of cells were performed according to a previously described method (White et al., 1996).

### Cell Proliferation Activity by Cell-Counting Kit 8 Method

HCT-8 cells in their logarithmic growth phase were digested with trypsin, collected by centrifugation, and resuspended in RPMI 1640 medium, and the cell count of the suspension was estimated. Following proper dilution, the concentration of the 2 cell suspensions was finally adjusted to 1 × 10^5^ /ml. Next, the cell suspension was inoculated in a 96-well microplate in an incubator for 24 h. After confirming that the cells were growing well, different concentrations of RES, VE, and RES + VE were added to the microplate. In addition, 5-FU (McLean, China) was used as the positive control for all experiments. Cell proliferation activity was determined using a CCK-8 kit (Beyotime Biotechnology, Jiangsu, China) according to the manufacturer’s instructions.

### Cell Scratch Test

When the cells were completely attached to the walls and covered the bottom of the well, the medium was discarded. A scratch was made using a 10 μl sterile pipette tip in each well. Next, the cells were washed 2–3 times with PBS. The cells (5×10^4^ cells/ml)were then cultured with different concentrations of RES and VE, and a blank control group (both serum-free medium) was set up (Xu et al., 2017). All cells were cultured in an incubator. Photographs were taken at 0, 12, and 24 h, and the percentage of healing of each scratch was calculated as follows:

$$Percentage\;of\;healing\left(\%\right)=\frac{scratch\;width\left(0h\right)-scratch\;width\left(others\right)}{scratch\;width\left(0h\right)}\times 100\%.$$

### Cell Cloning Assessment

The HCT-8 cancer cells were resuspended and counted, then 200 µl were cultured in an incubator for 24 h. Different concentrations of RES and VE were added to the cells and cultured for 24 h, after centrifugation which the supernatant of HCT-8 cancer cells was replaced with fresh medium and cultured for 14 days. After the culture, cells were washed with PBS twice and fixed with methanol for 15 min. Next, all cells were washed with distilled water twice, and then 1 ml of crystal violet working solution was added to each microwell and left to stand for 10 min at room temperature. The cells were then washed with distilled water twice and dried at room temperature.

### Flow Cytometry Detection

The cells were seeded in a 6-well plate with 2 ml of medium per well at 5 × 10^4^ cells/well. The cells were incubated in a 37°C, 5% CO_2_ incubator for 12 h to restore cell adherence. The culture medium was then discarded, and different concentrations of RES and VE (RES Group:12.5 μg/ml; VE Group:12.5 μg/ml; RES + VE, Low contration Group:6.25 μg/ml RES+6.25 μg/ml VE; RES + VE, High contration Grou: 12.5 μg/ml RES+12.5 μg/ml VE) or 0.6% ethanol were added to each well and cultured for 36 h. The annexin V-FITC staining of the cells was performed according to the manufacturer’s instructions (Beyotime Biotechnology, Jiangsu, China). Cells were harvested through trypsinization and washed twice with cold PBS (0.15 mol/L, pH 7.2). Subsequently, the cells were centrifuged at 1,000 × g for 5 min, the supernatant was discarded, and the pellet was resuspended in binding buffer at a density of 5.0 × 10^4^–1.0 × l0^6^ cells/ml. Thereafter, 100 µl of the sample solution was transferred to a 5 ml culture tube and incubated with 5 µl of annexin V-FITC (Beyotime Biotechnology) for 20 min at room temperature in the dark, after which 400 µl of binding buffer was added to each sample tube. The samples were analyzed using a CytoFLEX flow cytometer (Beckman Coulter, Brea, CA, United States).

### DAPI Nuclear Staining Determination

Two milliliters of culture medium containing 5 × 10^4^ cells was added to each well of a 6-well plate and incubated at 37°C and 5% CO_2_ for 12 h to restore cell adherence. After the medium was discarded, different concentrations of RES and VE (same contrations as mentioned above *Flow Cytometry Detection*) or 0.6% ethanol were added to each well, followed by addition of 0.5 ml of PBS to each well to wash the cells. Next, the cells were fixed with paraformaldehyde (0.5 ml, 4%) for 10 min, and incubated with 0.5 ml DAPI staining solution for 5 min. After the cells were washed thrice with PBS, their morphology was observed under the ultraviolet filter of an inverted fluorescence microscope.

### Expression of Apoptotic Proteins

Cells were sonicated for 5 min, and proteins were denatured by heating at 95°C for 5 min. Protein lysates (10–40 µl) were loaded onto 4–15% gradient protein gels and transferred to a polyvinylidene difluoride (PVDF) membrane. Membranes were saturated for 1 h and then immunoblotted overnight at 4°C with the indicated primary antibodies [Abmart Pharmaceutical (Shanghai) Co., Ltd., Shanghai, China] diluted in PBS. Anti-rabbit and anti-mouse antibodies conjugated with horseradish peroxidase (HRP) were used as secondary antibodies; these were diluted in PBS and incubated with the membranes for 1 h at room temperature. The infrared signal was integrated using an infrared imaging system. The band intensities were determined using the software associated with the chemiluminescence system.

### Statistical Analysis

All presented data are given as mean results of at least three independent measurements. Data are shown as mean ± standard deviation. Where it was necessary to compare three groups of data, a one-way ANOVA test was used. The results were considered significant if the *p*-value was <0.05.

## Results

**FIGURE 1:**
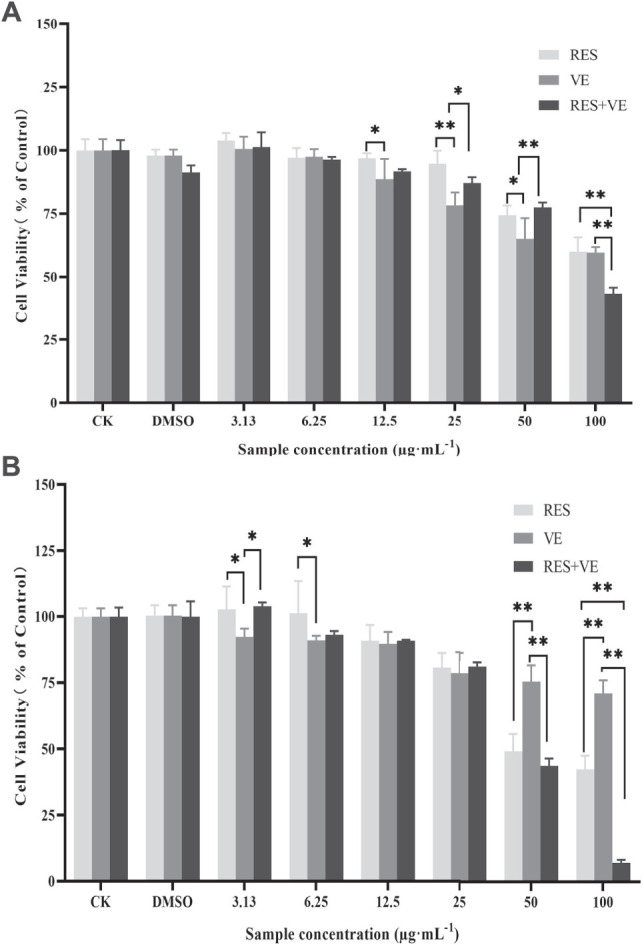
Effect of resveratrol (RES) and Vitamin E (VE) on HCT-8 cell proliferation: Cell-counting kit-8 (CCK8) assay 24 h **(A)** and 48 h **(B)**. Note: *p < 0.05, **p < 0.01, (Compared with the control group).

The inhibition test of active ingredients on cancer cell proliferation is the most effective way to evaluate the anti-cancer efficacy of active ingredients. The present study not only determined the effect of different RES and VE concentrations on HCT-8 but also investigated the effect of RES combined with VE on cell proliferation. Using the CCK8 assay, the cell proliferation activity was measured at 24 h (Figure 1A) and 48 h (Figure 1B) after treatment.

Compared with the control group, when the concentration of RES, VE or RES combined with VE exceeds 12.5 μg/ml after 24 h treatment, these groups can significantly inhibit cell proliferation (*p* < 0.05). when the concentration was greater than 12.5, RES, VE, or RES + VE groups significantly inhibited cell proliferation. At a concentration of 100 μg/ml, the effect of the RES + VE group was superior to that of the other groups (*p* < 0.01). As shown in Figure 1, the inhibitory effects of RES, VE, and RES + VE on HCT-8 cells had a dose-effect and time-effect relationship. The results of this study are consistent with those of other researchers on human mammary epithelial cells (Senthil Kumar et al., 2020), Caco-2 cell line (Huang et al., 2020), high-fat-diet-fed mice (Safahani et al., 2018). After 24 and 48 h of treatment, the combined effect significantly reduced the IC50 values of cell viability compared with single doses (*p* < 0.05) (Table 1), indicating that RES + VE inhibited the proliferation of cancer cells better than the single component group.

**TABLE 1:** Effects of RES, VE and their combination on the proliferation activity of HCT-8 cell.

Group	IC50 (µg/ml)	
	24 h	48 h
RES	175 ± 2.88^b^	70 ± 1.37^β^
VE	226 ± 3.56^a^	383 ± 3.08^α^
RES + VE	100 ± 2.39^c^	44 ± 1.15^γ^

**FIGURE 2:**
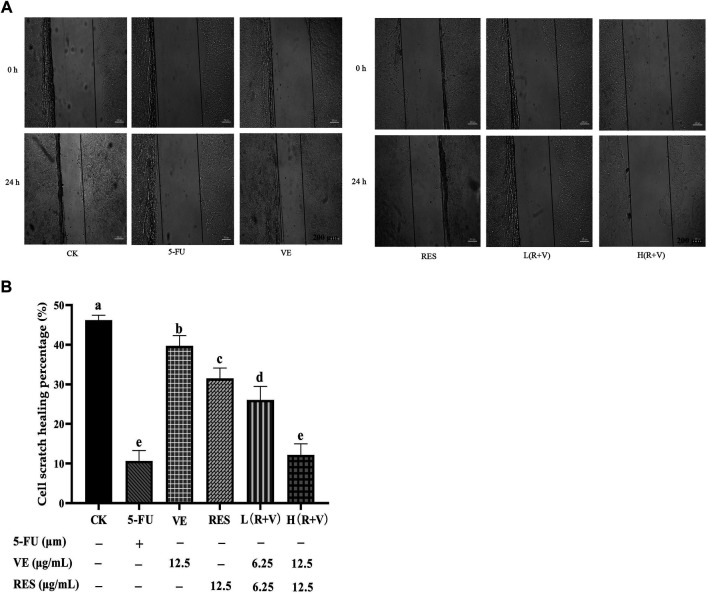
The effect of resveratrol (RES) combined with Vitamin E (VE) on HCT-8 cell migration ability after 24 h treatment. **(A)** 24- and 0 h comparison pictures of different treatments; **(B)** Migration rate after 24 h. Note: a, b, c, d, e represent the statistical results of different groups. Different letters represent statistically significant differences (*p* < 0.05).

Compared with the control group, the cell migration ability and scratch healing ability were significantly reduced after different treatments (*p* < 0.05) (Figure 2). The scratch healing ability of the low-dose combination group (6.25 μg/ml) was lower than that of the single component group (12.5 μg/ml) (*p* < 0.01) (Figure 2B). Compared with the low-dose group, the high-dose group (12.5 μg/ml) and the positive control (5-FU) significantly reduced the scratch healing ability (*p* < 0.01) (Figure 2B).

**FIGURE 3:**
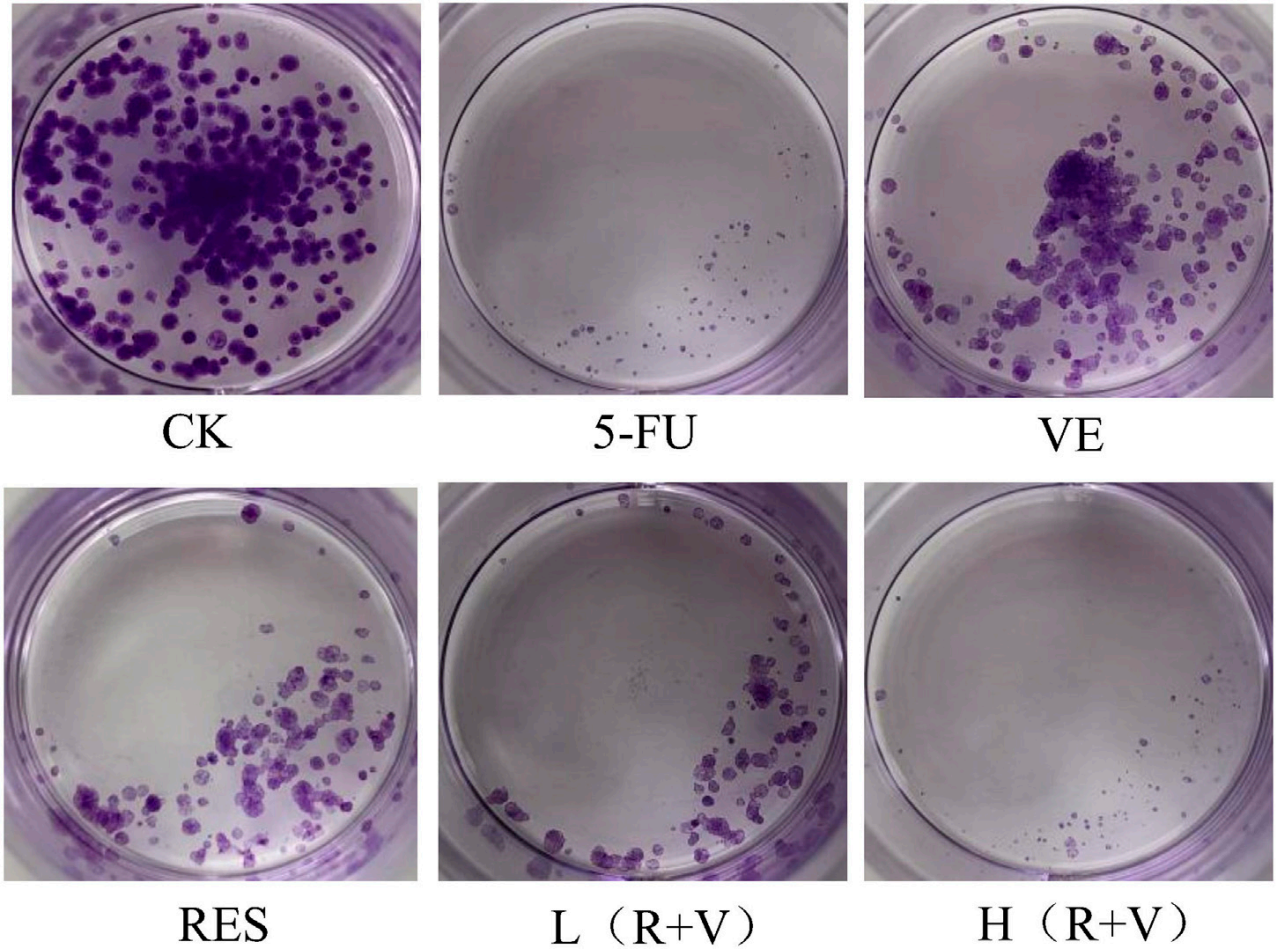
The influence of resveratrol (RES), Vitamin E (VE), and their combination on cell clone formation.

Compared with the control group, the number of HCT-8 cell colonies and cells in the single-component group and the low-dose combined treatment group were reduced (Figure 3). Thus, the high-dose treatment group, like the 5-FU treatment group, can significantly reduce the cell population.

**FIGURE 4:**
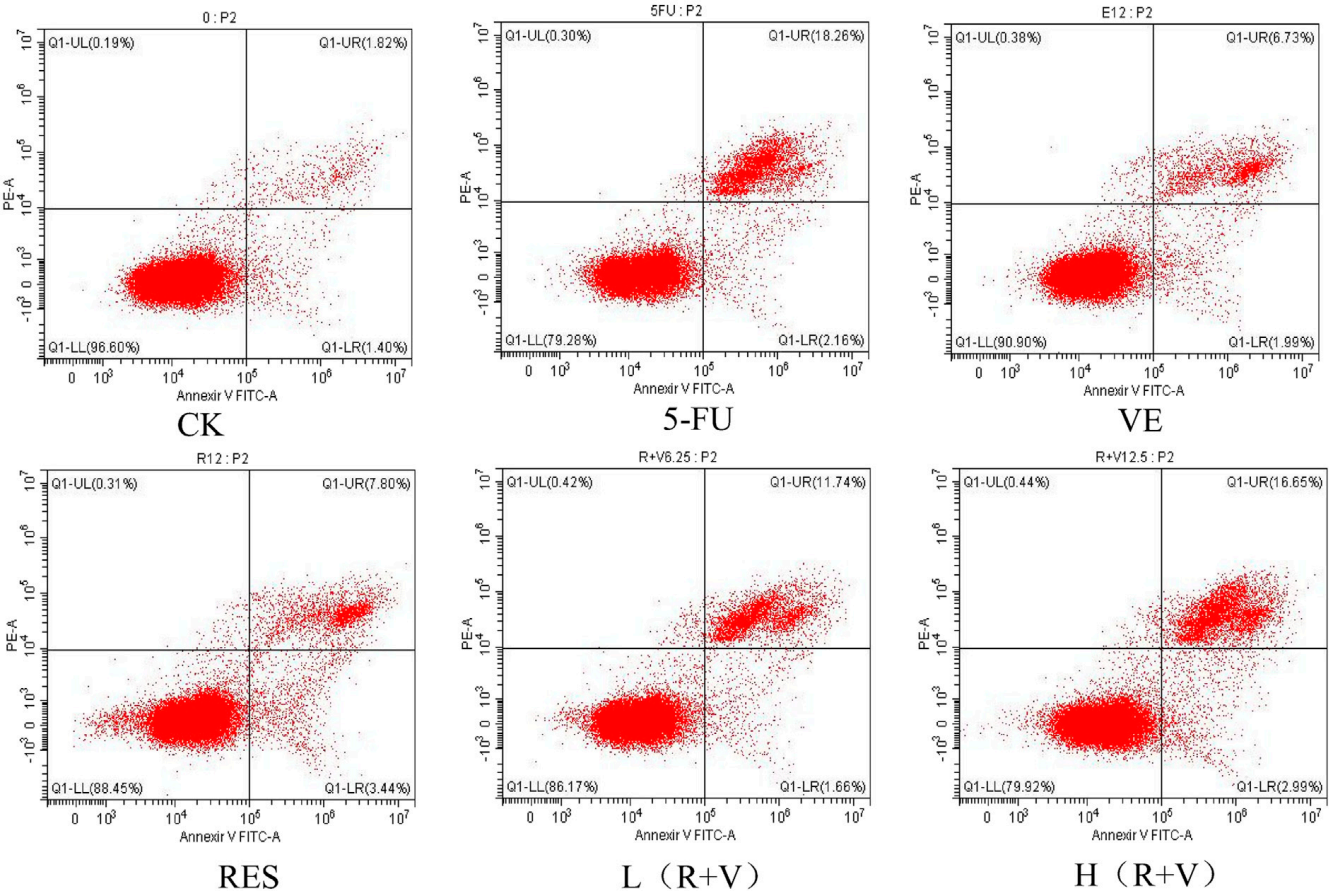
Flow cytometry to determine the effects of different treatments on cell apoptosis.

**FIGURE 5:**
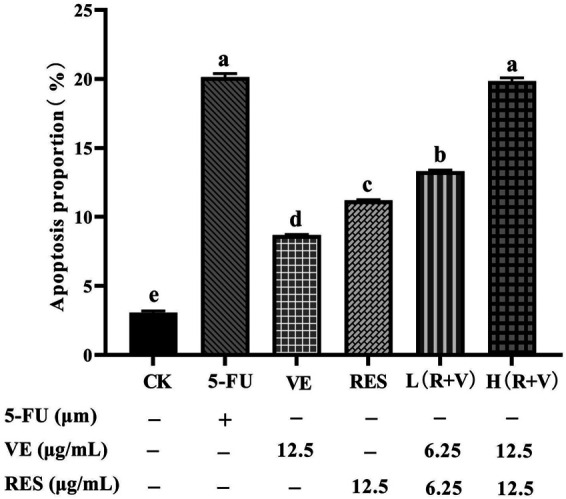
The effect of different treatments on cell apoptosis. Note: a, b, c, d, e represent the statistical results of different groups. Different letters represent statistically significant differences (*p* < 0.05).

Compared with the control group, the samples can significantly induce early and late apoptosis of the HCT-8 cells (Figure 4). The apoptotic rate of the control group was 3.07 ± 0.13%, and those for the 5-FU, VE, RES, Low-dose RES and VE group [L (R + VE)], and High-dose RES and VE group [H (R + VE)] were 20.13 ± 0.25%, 8.69 ± 0.30%, 11.2 ± 0.43%, 13.30 ± 0.11%, and 19.84 ± 0.24%, respectively (Figure 5). The apoptotic rate of the combined treatment group was significantly increased (*p* < 0.05), and the H (R + V) group did not significantly differ from the positive control group in enhancing colorectal cancer cell apoptosis (*p* > 0.05). This shows that the combined treatment had a good synergistic effect, and its effect on enhancing colorectal cancer cell apoptosis is similar to that of the positive control.

**FIGURE 6:**
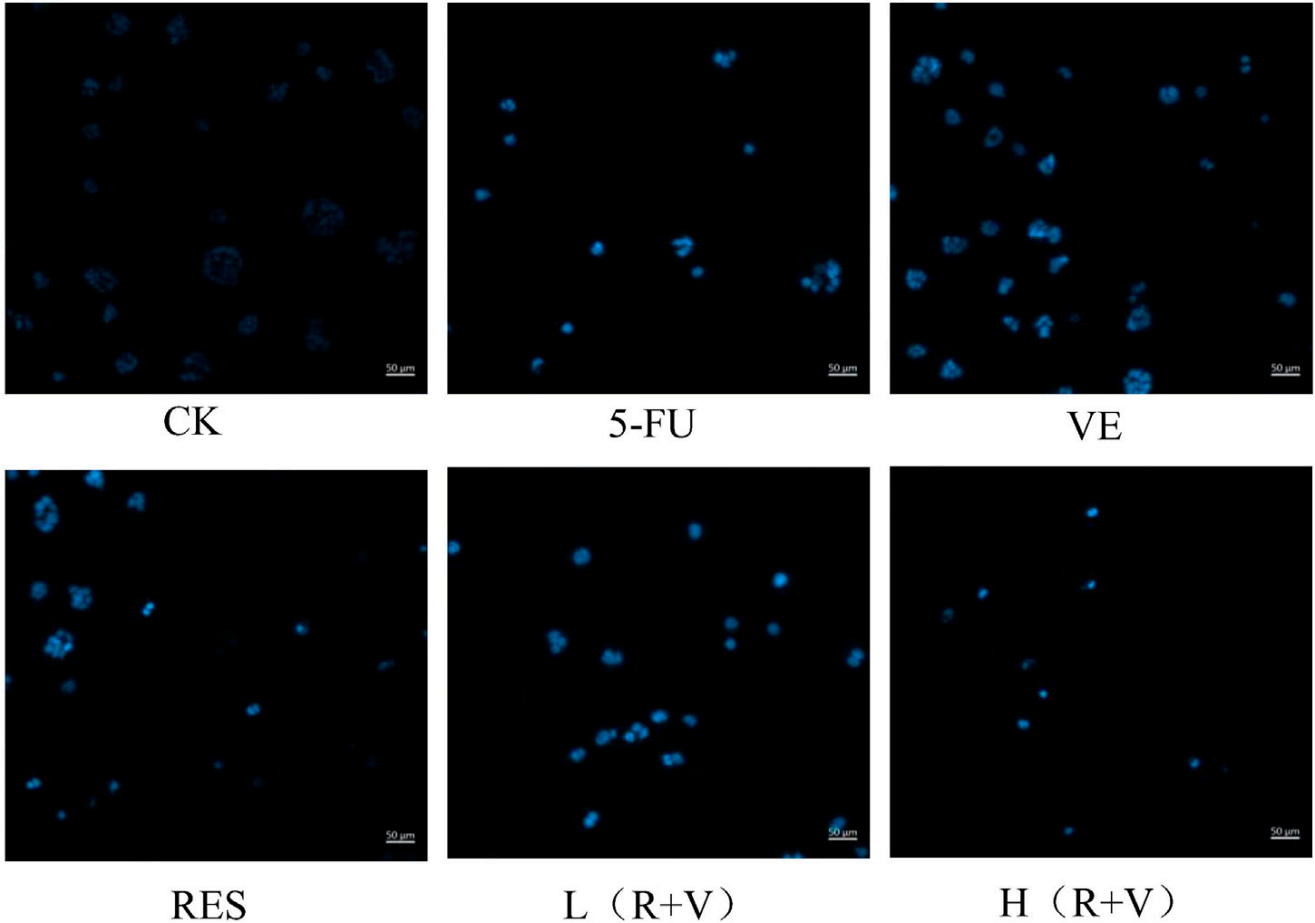
Morphological changes of HCT-8 cell nuclei under an inverted fluorescence microscope after 36 h of treatment with different drugs.

DAPI is a blue fluorescent, nucleus-specific dye that can be used to stain live or fixed cells. The dye has minimal fluorescence in solution but becomes brightly fluorescent upon binding to DNA. After 36 h of treatment, the morphological changes of HCT-8 cell nuclei were observed under an inverted fluorescence microscope (Figure 6). Most cells in the control group were smooth polygonal and stained light blue upon dying with DAPI, and they were excited by UV light. In contrast, after different treatments, the HCT-8 cells had shrunken nuclear chromosomes and stained bright blue. Furthermore, compared with the single sample groups (VE and RES groups), chromosome pyknosis was more evident in the combined treatment groups [L (R + V) and H (R + V) groups], with dense, bright blue staining. This result is consistent with that of apoptosis observed by flow cytometry, further indicating that the combined action can significantly promote cell apoptosis.

Studies have reported that RES (Jeong et al., 2015; Buhrmann et al., 2018) and VE can regulate and promote colorectal cancer cell apoptosis through apoptosis-related pathways. Therefore, in order to further study the mechanism of RES and VE in promoting colorectal cancer cell apoptosis, western blotting was performed to detect the expression of BCL-2, BAX, caspase-3-, caspase-8-, and caspase-9-related proteins after RES and VE intervention.

**FIGURE 7:**
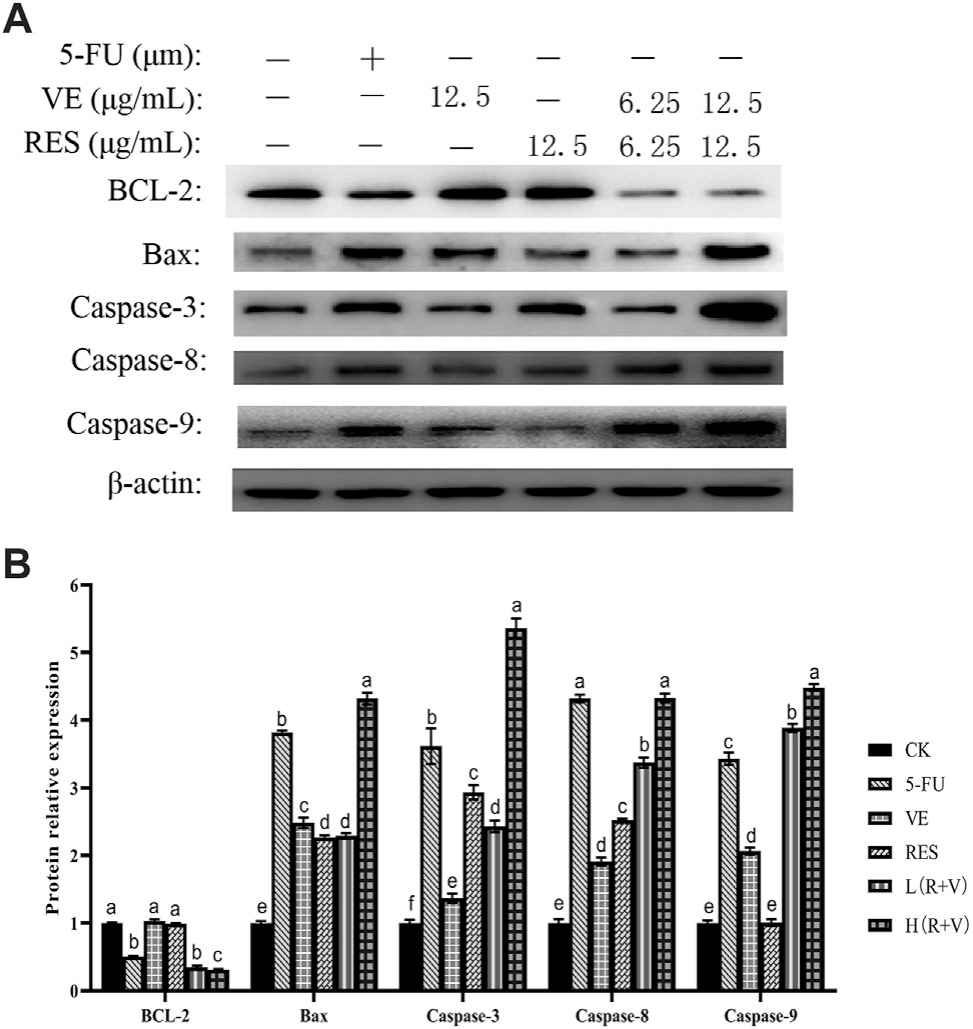
Changes of apoptosis-related protein expression in HCT-8 cells after different treatments. **(A)** Western blot results of apoptosis-related protein expression; **(B)** Protein expression levels in HCT-8 cells treated with different drug groups.

In the expression of the anti-apoptotic protein BCL-2, the combined action group [L (R + V) group, H (R + V) group] significantly downregulated the expression of the anti-apoptotic protein (*p* < 0.01) (Figure 7); however, there was no significant difference between the L (R + V) and 5-FU groups (*p* > 0.05). Compared with the 5-FU group, the expression of the anti-apoptotic protein in the H (R + V) group was significantly decreased (*p* < 0.05).

The above results indicate that the combined effect of RES and VE can significantly downregulate the expression of anti-apoptotic proteins (*p* < 0.05) and significantly upregulate the expression of pro-apoptotic proteins (*p* > 0.05), which is comparable to the effect of the positive control.

## Discussion

RES and other active ingredients can have an improved synergistic promotion effect. Lee and others (Lee et al., 2015) investigated the anti-angiogenic effects of RES and 5-FU either alone or in combination in a B16 murine melanoma model. They found that co-treatment inhibits cell proliferation more efficiently than either drug alone. Research on gastric cancer also proved this dose-dependent relationship of RES. Treatment with RES (12.5–200 μmol/L) for 48 h significantly inhibited colorectal cancer stem cell (CCSCs HCT116) proliferation by increasing the proportion of cells in the G0/G1 phase and decreasing those in the S phase in a dose-dependent manner (Rauf et al., 2018). In the present study, the effective concentration of the VE group was reduced to 3.13 μg/ml in the 48 h treatment group. Compared with the 24 h treatment, the effective concentration of the RES + VE group was reduced to 50 μg/ml in the 48 h treatment. Both RES and VE can significantly change the antioxidant metabolism, so we hypothesize that they have a synergistic effect in anti-cancer. These results indicated that RES and VE have a significant synergistic inhibitory effect on cancer cells relative to a single ingredient.

At higher/anti-angiogenic concentrations, RES inhibits HUVEC tube formation and cell migration/invasion (indices of angiogenesis) (Wong and Fiscus, 2015). In addition, it inhibits phorbol 12-myristate 13-acetate (PMA)-induced invasion and migration in both A549 and HeLa cells (Kim et al., 2012). These studies demonstrated that RES inhibits LPS-induced tumor migration and EMT markers and significantly extends animal survival in addition to reducing the tumor size (Chen et al., 2012). All results indicated that RES has a good ability to inhibit cell migration. In this study, the cell migration ability was assessed by the cell scratch image and the scratch healing percentage value. Literature studies (Chen et al., 2012; Kim et al., 2012; Wong and Fiscus, 2015) have shown that the anticancer effect concentration of RES alone is at least greater than 100 µM. The results of this study show that the effect concentration of RES or VE alone is 12.5 μg/ml (about 55 µM). This may be related to different cancer cells. However, when RES and VE work together, only 6.25 μg/ml is required to be effective. Therefore, the synergy of RES and VE is very obvious.

The cell clone formation rate is the cell inoculation survival rate, which indicates the number of adherent cells that survived and formed clones after cell inoculation. The adherent cells may not be able to proliferate and form clones, but the cells that form clones must be adherent and proliferating cells. Thus, the clone formation rate reflects the two important traits of cell population dependence and proliferation ability. Our results showed that the combined effect of RES and VE could significantly inhibit colorectal cancer cell proliferation.

Apoptosis is a type of self-regulation that controls cell growth and homeostasis. However, when apoptosis is dysregulated, it results in the uncontrollable growth of cancer cells and induces various cancers. Therefore, inducing apoptosis is an effective way to kill cancer cells.

## Conclusion

The RES combined with VE group significantly inhibited the *in vitro* growth and proliferation of HCT-8 colorectal cancer cells and reduced the IC50 value in comparison with the single component group. In addition, the RES combined with VE group had a greater effect on the alteration of cell morphology, cell colony formation, significantly reduced cell migration ability, and significantly induced apoptosis in colorectal cancer cells compared with the single component group (*p* < 0.05). Importantly, these effects were comparable to those of the positive control.

The combined effect group of RES and VE could significantly upregulate the expression of pro-apoptotic proteins BAX, Caspase-3, Caspase-8, and Caspase-9 and downregulate the expression of anti-apoptotic protein BCL-2 compared with the single component group, thus demonstrating that RES and VE synergistically promote apoptosis in colorectal cancer cells effectively.

## Data Availability

The original contributions presented in the study are included in the article/Supplementary Material, further inquiries can be directed to the corresponding authors.
